# The International Dental Congress

**Published:** 1904-10

**Authors:** 


THE AMERICAN
JOURNAL OF DENTAL SCIENCE

### THE INTERNATIONAL DENTAL CONGRESS.

The International Dental Congress held in St. Louis was attended by representative dentists from almost every County, there being in attendance probably two thousand dentists.

Dr. H. J. Burkhart of Batavia, N. Y., was elected president, Dr. B. Holly Smith of Baltimore making the nominating speech.

Dr. Rodriguez Ottolengui of New York placed in nomination for the same office Dr. C. N. Johnson of Chicago, but the latter withdrew.

Dr. E. C. Kirk of Philadelphia was chosen secretary over Dr. A. W. Harlan of New York. Dr. M. F. Finley of Washington, D. C., was chosen treasurer without opposition.

This business having been disposed of, addresses were made by Dr. C. C. Chittenden of Madison, Wis., president of the National Dental Association; Dr. Burton Lee Thorpe of St. Louis, for the dentists of Missouri; by Dr. A. W. Harlan of New York, for the International Dental Federation, and by Dr. Otto Zisgmondy and Dr. R. Weiser, both of Austria, for the foreign delegations.

It might almost be said, that the interest in the Congress as a Congress, died at the adjournment of this session. Thereafter the meetings followed the thoroughly well known and recently abandoned system which so long has marred the National Dental Association.

There were morning sessions of the main body, before which a few papers were read, selected by no previously announced rule. The afternoons were given over to meetings of the sections, and if the ignominious failure of this method of managing a dental meeting, which must be recorded in connection with this Congress, would only result in its final abandonment in this country, at least one great good will have been achieved by this Fourth International Dental Congress, which was International in character, only at one session.

Let us mention a few of the unpleasant features of working by sections, and then discuss the reasons therefor. It must first be recorded, however, that it was reported that no less than sixteen hundred dentists joined the congress. Just how many actually attended was not made known, but probably a thousand; possibly more. Let these figures be borne in mind. The meetings of the section on operative dentistry were held in the main assembly room, and whether for this reason, or not, the sessions were fairly well attended, as many as one hundred being occasionally present. The prosthetic section had trouble from the start, its very first session being adjourned for lack of an audience. On other days the attendance fluctuated between five and forty, the latter being a generous estimate. Yet surely this section, dealing with the every day work of the dentist might reasonably have looked for greater support. The same list of papers, if offered at almost any State society meeting would have attracted from two to five hundred men, according to the locality. The Odontographic Society of Chicago with only five papers, two years ago, had twenty-six hundred.

There were so many delays, waiting for auditors, and the sessions for the same reason were so short, in this section, that at the last there were several important papers by men from abroad, all of which were crowded into one slimly attended session, and read so hurridly that discussion perforce was cut off.

Think of the feelings of the gentlemen who crossed the ocean to read papers, when they found themselves in this great American land, among the men that boast so of American dentistry, to be met, as these were by less than twenty auditors? But there was worse even than this. Dr. Guerini, of Italy, brought into this section several exceedingly interesting examples of prosthetic work, which he desired to show and explain, such as swaged aluminum plates; gold plating on aluminum; soldered aluminum; an exceedingly ingenious spring for use in connection with obturators; but Dr. Guerini never was reached at all, and as he expressed it, he "packed his things and took them back to Italy." It became the painful duty of the section officers to apologize for that, for which there was no excuse, and this was all the more difficult as this same gentleman had brought a paper which he had been especially requested to prepare; a paper which must have cost him endless work, as it included a resume of the literature of dental art in Italy; nevertheless, Dr. Guerini complained that he had on two different days repaired to the proper section with his paper only to find no one in attendance. This circumstance was brought to the attention of Dr. Burkhart, and subsequently opportunity was afforded Dr. Guerini to read this paper.

Dr. E. Sauvez, of Paris, not being able to speak English as fluently as he would have wished (though quite to the satisfaction of the Americans who had the pleasure of hearing him in an after-dinner address at the banquet of the Interstate Dental Fraternity), had his paper not only translated into English, but he assumed the expense of printing it in pamphlet form, bringing with him for distribution among the members, two or three thousand copies. The subject was certainly one of vast interest, "Local Anaesthesia." Yet in spite of the importance of the subject, in spite of the distance he had traveled, in spite of the fact that he was our guest and we were presumably playing host, when the time for reading his paper arrived just four men were present in the section.

But even this record was beaten. Our own Dr. Cryer, whose papers are always fine, brought to this Congress, one in connection with which he used the lantern and showed the finest radiographs of jaws ever seen, including some that were stereoscopic in character. Yet Dr. Cryer was allowed to sit for an hour in his section in the sole companionship of the gentleman in charge of the lantern and the stenographer. Finally, Dr. Ottolengui passed through the room, having been previously engaged in the orthodontia section reading his own paper, and Dr. Cryer invited Dr. Ottolengui to preside, while he read his paper, and this Dr. Ottolengui did, esteeming himself much honored to be the whole audience before which Dr. Cryer was willing to deliver his lantern lecture.

An auctioneer sometimes begins a sale with none present but his own clerks, hoping that the sound of his voice may attract the passerby. And thus it was in this instance. The pictures on the screen caught the eyes of a few stragglers, until a full dozen were present. But as soon as discussion was called, they faded away, and then it was seen that the visitors had been mere gallants with their dames, seeing the sights. They were not dentists at all. Yes, there was one, just one left out of the phantom dozen, and he and Dr. Ottolengui discussed the paper. Was this incident far short of being a disgrace?

Other splendid papers brought over by distinguished foreigners were not even offered, when the authors saw how small their audiences would be.

The orthodontia section was practically a success, though even here the attendanc never was large. The success here was due to the presence of the members of the

American Society of Orthodontists, who gave almost all their time to this one section. Why? Because orthodontia is the only dental specialty which is really an organized separate speciality of dentistry, and therefore orthodontia is the only field of work which can fittingly be represented by a section.

This brings us to a consideration of the reasons for the failure of the sections. These may be divided into two classes, under the nominations, bad management, and bad method, the latter undoubtedly predominating. Even with good management this section scheme must have shown poor results, though undoubtedly better.

The Fourth International Dental Congress, presumably was projected as a convention at which the dentists of the world might exchange views on subjects relative to their calling. It primarily resolved itself into the finest display of dental goods the world has ever seen. This great show, was continuous, open uninterruptedly from sunrise to sunset, and if there were a thousand dentists at the Congress, about eight hundred were in the exhibit rooms all the time. This left about two hundred to visit the meetings of the ten sections, taking no count of those that went to the World's Fair. Let us pause here to take a note. The World's Fair buildings were closed every evening; thus those that wished to see their interiors were obliged to go during daylight. Yet, the Congress did not have a single night session.

To return to the matter of the exhibits, it has been explained that the exhibitors refused to take space unless permitted to remain open continuously, and at the time of making contracts with them it seemed essential to have them—and their money. There is no doubt that exhibits are an important item in making a meeting successful, and we must not be misunderstood as arguing against exhibits, but we do think that the exhibition of dental goods should be subordinate to the reading and discussing of scientific papers, more especially when we are supposed to be receiving guests from foreign lands. Moreover, we think the end might have been attained, even though it were necessary to give the exhibitors all day in which to show their wares.

Another bit of mismanagement which militated against success of the sections, was the absence of bulletins. A large board at the entrance, containing official and correct announcement of each day's doings, would have helped mightily.

But, after all, the main trouble lay in the system. Seemingly, because medical conventions have sections, it has been thought necessary for dentists to conduct their larger meetings in similar fashion. But there is no logical analogy. The medical world is finally split into definite fields of work, fields in which the worker is exclusively occupied with[illegible]
tations. A section in surgery attracts the surgeon. A section on gynecology, the gynecologist. A section on chemistry, the chemist, etc., etc. It is quite different in dentistry. The sub-division into specialties is in its infancy. Orthodontia is the only specialty in dentistry, which at present has any real separation, and it is this fact alone which made the section in the Congress devoted to this subject more suc-
dentistry. Yet there are probably not more than fifty orthodontia specialists in the whole United States. In the present state of affairs, then, we should recognize that dentistry is just dentistry, and that the dentist is interested in all that pertains to his work. If imaginary lines be drawn, and men are forced to work in fields separated from the general theme, the workers will be few, even though the work itself may be better; and for like reasons the audiences will be slim.

Viewing a programme divided into ten sections, the plain dentist finds himself at a loss to know which room will offer the most pabulum. He may find several papers in different sections which he would particularly desire to hear, and should he attempt to be present, most likely he will find them all called for the same hour. An actual fact sometimes points a moral better than any argument. A certain dentist had been asked to open the discussion on three papers at this Congress. In each instance his name had been sent to the committee by the essayist; thus each essayist thought this particular man qualified to discuss his paper. It chanced that all three papers were called for exactly the same hour, in three different sections. Of course it was physically impossible for him to be in three places at once, and this is the turning point of the argument. It should not be a physical impossibility for any member of a Congress to hear all of the papers. On the contrary it should be made possible for every man to hear every paper.

It would be manifestly wrong to criticise, without showing a means of improving the method. There is, of course, some good to be attained by section work. Without going too deeply into this phase of the subject, suffice it to say that it assures a diversity of topics. At one time the American Dental Association, worked in sections which were nominal only. Papers were read by title in the sections, and then read in full before the main body. This insured an audience, but it allowed one or two sections to monopolize a whole meeting. The method at the present Congress was the reading of papers before the sections, and each section was obliged to attract its own audience. The evils of this have been, in part, pointed out.

If it be deemed wise to work by sections in the present status of dentistry, the divisions should be as broad as possible. The three section plan, recently adopted by the National, will surely be better than ten. But the reading of papers in sections, and especially if the various sections meet simultaneously, must prove disastrous by dividing the interest.

Let us suppose that in the last Congress there had been three sections, sub-divided if necessary into three parts, or sub-sections, it being understood that these sections and sub-divisions were made merely for the purpose of systematizing the matter to be obtained and offered at the Congress. Suppose a rule requiring all papers to be in the hands of a committee for consideration at least one month prior to the opening of the Congress.

On the morning of the first day, the organization of the Congress. In the afternoon, meetings of the three sections, each to receive the reports of the essay committees; such committees to report all papers received, and to recommend, first papers to be rejected; second, papers to be read by title only, and published in the transactions; and third, papers to be presented to the Congress. As one argument for this part of the method, be it said that much valuable time was spent on papers which should have heen read by title only; while some of the best papers were read by title for lack of time, or lack of attendance. On the evening of the first day, the Congress would have been placed in fine condition for real scientific work. This session should have been devoted to papers from Section I. The mornings might have been free, and a meeting of the full Congress could have occurred each afternoon and evening, excepting one evening for the banquet. The sections could have been taken up in rotation (according to this plan where would have been but three sections). Each section could have had two meetings, before the full body. There would have been ample time for clinics. The exhibits would not have noticeably interfered and what is equally important, since thus far all our Congresses have been adjuncts of World's fairs, the mornings spent visiting the Fair, would not have taken the men from the scientific meetings.—*Items of Interest.*

---

The following were elected as the American representatives on the excutive council of the International Dental Federation: Dr. Burton Lee Thorpe, Dr. B. Holly Smith, Dr. Wm. Carr, Dr. A. W. Harlan, Dr. E. C. Kirk.

The next congress will meet in Berlin in 1909. Dr. W. D. Miller will probably be made the president.

---

The new officers elected for the National Dental Association were, president, Dr. W. E. Boardman, Boston; vice president from the east, Dr. John I. Hart of New York; vice president from the south, Dr. B. F. Lucie of Hill Spring, Miss.; vice president from the west, Dr. Wm. Conrad of St. Louis; secretary, Dr. A. H. Peck of Chicago; corresponding secretary, Dr. C. S. Butler of Buffalo; treasurer, Dr. V. S. Turner, Raleigh, N. C.

The Fourth International Dental Congress has passed into history, and glorious history it has made. Never in the history of the profession were so many great and famous men gathered together, never were so many really great papers read and discussed, and never was there a better clinical demonstration of methods and ideas. Time and space would fail me were I to attempt a resumé of the entire congress, were I to describe the notables there, the Grand Old Man of dentistry, Dr. G. V. Black, honored and revered by all for his epoch-making labors in the advancement of his profession, and loved by all those who have had the rare privilege and honor of his friendship; Dr. Miller of Berlin, the great bacteriologist of the profession, and hosts of other stars of the first magnitude. Neither can I attempt a synopsis of the papers read, but it will amply repay the members of the profession to carefully study them when they appear in the transactions. Nor can I give even a glimpse of the business meetings of the congress, nor describe the officers who carried off the honors of the congress so efficiently. I can but tarry a brief moment over that part of the congress, that judging from the attendance at its sessions, had the greatest interest for by far the large majority of the members of the congress. I refer to the clinics. The clinical program was carried out in the large gallery of the exposition hall of the great exposition building of St. Louis. The large gallery entirely encircled the great building and gave ample room for all the clinics. A large skylight, running the entire length of the room, furnished an abundance of light. There were operations of all kinds, extractions, fillings of all kinds, porcelain inlays, etc., etc. But again I must confine myself to but a small part of this portion of the congress. A great deal of interest was manifested in the very creditable inlay operations that were made, and porcelain undoubtedly made many friends. But the section of all sections that called forth the loudest encomiums of praise was the section devoted to the Black Club, who were operating under the direction and demonstrating the teachings of that greatest of Dr. Black's pupils, Dr. E. K. Wedelstaedt of St. Paul. The praise that was bestowed upon each operation—and they were endless—was acknowledged by the operator to belong not to the individual making the operation, but to the man who has unceasingly and by making the greatest personal sacrifices labored among the men of the Northwest, for the sole purpose of advancing the art of dentistry and teaching the principles of Dr. G. V. Black. In inspecting the operation of the men of the Black Club one was struck with the similarity of method and technique. As we passed from chair to chair the same method of cavity preparation was observed, the same definite manner of introducing and condensing the gold, and the same method of finishing and polishing the filling, so that one was impressed with the thought, which soon became conviction, that these men were the disciples of one man and that man had a method which he had been capable of thoroughly impressing upon his pupils, so that the finished filling of one man looked as though it might have been made by any other man in the club, and each filling as near perfect as it was possible to make it. One of the greatest educators in the world said, "There are not fourteen other men in the world that could make such operations." Another, almost equally well known, said to the writer, "Those men in the Northwest are making the greatest operations that are being made anywhere in the world." And he added, "It is all due to the teaching and the self sacrificing labors of Dr. E. K. Wederstaedt." Those of us who know something of the great work which Dr. Wederstaedt has done, and is doing, rejoice at the great triumph which he won there. But knowing the modesty of the man, we also know that this is a secondary consideration with him. If the demonstration of the methods which he has been teaching shall cause men to pause in their unscientific methods of operating upon the human teeth, and shall cause them to study conditions, and then with an intelligent knowledge of the structure of the teeth, the causes of their decay, and a knowledge of the physical properties of those materials used for filling the teeth, shall so operate that humanity shall be benefited thereby, he will feel that his labors have been repaid. All honor to Dr. E. K. Wedelstaedt.

It was a great pleasure to meet the representatives of our profession from foreign fields—the men from England, Germany, France, Spain, and other of the old world countries, and men from the southern continent, from the islands of the sea, and from all parts of our own fair land. May the Fourth International Dental Congress be but a small beginning of the many great international congresses that shall follow in the years to come, and may this meeting together of professional brethren strengthen the brotherhood of nations so much that it shall be a potent factor in causing wars to cease, and internal social questions to be relegated to the rear.

J. V. Conzett, D. D. S.
*Dubuque, Iowa.*

